# Increasing access to a global health conference and enhancing research capacity: Using an interdisciplinary approach and virtual spaces in an international community of practice

**DOI:** 10.7189/jogh.12.03038

**Published:** 2022-07-06

**Authors:** Francesca W Basile, Jaime Petrus, Catriona Gates, Sarah H Perry, Jennifer Benjamin, Kevin McKenzie, Kajal Hirani, Cam Huynh, Florence Anabwani-Richter, Heather Haq, Diane Nguyen

**Affiliations:** 1 Baylor College of Medicine International Pediatrics AIDS Initiative (BIPAI) at Texas Children’s Hospital, Houston, Texas, USA; 2 Department of Pediatrics, Baylor College of Medicine, Houston, Texas, USA; 3 Department of Education, Innovation, and Technology, Baylor College of Medicine, Houston, Texas, USA; 4 Baylor College of Medicine Children’s Foundation-Eswatini, Mbabane, Eswatini

The foundational principles of global health (GH) organizations are rooted in the recognition and amelioration of inequities that result in poor health. Despite capacity enhancing strategies designed as bilateral partnerships with colleagues in low- and middle-income (LMIC) settings, international organizations from high-income countries (HIC) still struggle to address the lack of opportunities for professional development amongst LMIC partners.

The pursuit of equitable representation in GH meetings is crucial to capacity enhancing, as there is more to a conference than networking and knowledge transfer; presentation at a conference is often a defining career development stepping-stone. However, most international conferences favour the participation of persons from HIC by being held in expensive locations, frequently with strict visa requirements hindering inclusive engagement. Authors additionally face competitive abstract selection processes that may constitute barriers for junior colleagues from LMICs who often lack essential resources and mentorship; however, these are the very individuals principally involved in programs at the ground level. As a result, GH conference attendance and participation are skewed towards representatives from HIC [1]. The SARS-CoV2 pandemic presented an opportunity to re-imagine possibilities and eliminate these barriers [2-4]. We describe our efforts to address disparities and utilize technology to introduce a wide-reaching educational initiative.

## THE RAISE SYMPOSIUM

The Baylor College of Medicine International Pediatric AIDS Initiative (BIPAI) Network includes nine local and independent partner organizations (Foundations) that provide paediatric and maternal health care for vulnerable populations across sub-Saharan Africa, South America, and Eastern Europe. Collectively, these Foundations employ nearly 2600 local staff serving over 365 000 patients in their communities. Texas Children’s Hospital (TCH) and Baylor College of Medicine (BCM) provide the Network with technical assistance and capacity enhancing opportunities in partnership with each Foundation.

**Figure:**
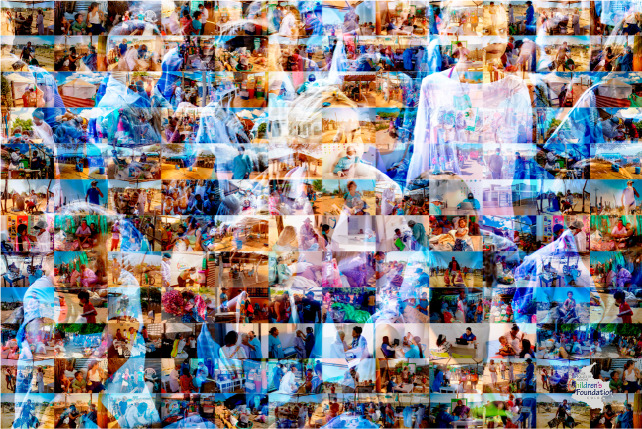
Photo: RAISE Symposium award-winning photography ‘The Photographer’s eye’. In addition to the call for scientific abstracts, BIPAI network staff and patients were invited to submit their artwork. The Symposium attendees voted using QR codes on their favourite submissions from several categories (photography, jewellery, drawings/paintings, sculpture, textile, crafts). Source: David Brito Ortega. Used with permission.

Historically, BIPAI has hosted an annual in-person meeting for an average of 100 people, 70% from the Foundations and 30% from TCH/BCM. BIPAI sponsored approximately five individuals from each Foundation, resulting in an exclusive meeting not available to most Foundation staff. BIPAI re-evaluated this in-person meeting model in light of the SARS-CoV2 pandemic. Online education has been suggested as a long-term strategy for achieving equitable progress in GH if barriers such as unreliable internet connectivity, time zone differences, and lack of meaningful attendee engagement are addressed [5]. We perceived the accessibility of the virtual format as an opportunity to expand the participation of staff who had been previously excluded from the in-person meetings.

With this vision in mind, we conceptualized and piloted a virtual event, the RAISE Symposium, in July 2021. We built the Symposium around five thematic areas (RAISE: Research, Art, Innovation, Scholarship, and Education), designing each session to be highly interactive and usable, nurturing scientific curiosity and abilities of local staff.

To foster collaboration and input from the global community, we chose to host the live event on Zoom as it is a free online platform, with minimal bandwidth requirements and accessibility from smartphones. We preferred the “meeting” over “webinar” option to allow for more interaction and inclusiveness. We chose VoiceThread©, a multimedia-based, virtual collaboration platform that allows users to upload, share, and discuss videos and presentations, as the venue for e-posters and art exhibits. Organizers and participants could post written or audio comments and questions on posters, with easy access through a computer or mobile device. Virtual discussions developed organically and enabled stimulating conversations that promoted collaboration and networking.

## THE RAISE FRAMEWORK: COLLABORATIVE SCOLARSHIP AND MENTORSHIP

Collaborative scholarship is a BIPAI core value that demands innovative approaches to improve access to professionals across disciplines. The GH Scholarship Community of Practice (CoP) is a pre-existing network-wide program fostering a culture for scholarship through enhancing fundamental scholarly skills and building community. The CoP helped establish the virtual event, providing the educational framework for a supportive learning event where participants could practice their scholarly skills, showcase their work, and allow for true knowledge transfer [6].

We integrated resources by providing coaching and educational activities facilitated through CoP and offered a variety of topics and activities to encourage both active and passive engagement of staff other than clinicians and researchers. The goal was to catalyse the exchange of ideas across the Network beyond the traditional abstract showcase, making scientific and programmatic research more accessible and appealing. The Symposium was organized through eight months, the majority of which was dedicated to coaching activities for submitting authors and foundations.

All individual submissions underwent a double-blinded peer review process and were accepted into three categories (oral presentation, live e-poster, e-poster) based on scientific merit. We identified 46 expert reviewers across the Network through an online survey and matched them with abstracts meeting their scientific interests. Three independent reviewers assessed each abstract. Reviewers used scoring rubrics to guide evaluations and provided a numerical score and anonymous written feedback to the authors.

Comprehensive coaching support, facilitated by CoP satellite activities, was the primary capacity enhancing initiative incorporated into every step for preparing the Symposium. The immediate goal of coaching was to support preparations of scientifically robust abstracts for submission, support the development of effective and engaging posters or presentations, while enabling authors to present their work. We also intended for coaching to enable ongoing collaborative or mentoring relationships through the CoP; this would continue to foster the scholarship-rich culture beyond the event.

Prior to the abstract submission deadline, we paired 29 authors from various Foundation sites with 20 experienced volunteer faculty members with expressed interest and experience in GH. They provided advice for 37 abstracts related to content, research design, statistical analysis, and writing. We accepted 23 abstracts from 19 authors who had received coaching for presentation (2 oral, 7 e-poster and 14 poster presentations). More than 90% of authors reported gaining skills transferable to future submissions, with our coaching encouraging iterative processes for the long-term improvement of their work. Authors selected for oral presentations joined an hour-long CoP satellite session about effective oral communication; it introduced the concept of 5-minute presentations and the creation of engaging slide decks. Attendees reflected that they “felt more confident mastering a short oral presentation.”

Recognizing the need for standardized and novel poster designs to improve quality and communication of key results, we embraced the “#betterposter” movement [7-9]. We offered three poster templates (one “traditional” and two innovative visual formats) and facilitated a CoP satellite session to encourage the use of infographics. The impact of this initiative was tangible, with 73% of e-posters presented at RAISE using novel formats, and 78% containing at least one infographic.

To highlight the potential of cross-network discussions, we invited authors of closely aligned scientific abstracts to co-present at the Symposium. Joint presentations connected authors and allowed the audience to learn dynamically and be inspired by the potential of network-wide collaboration. For example, one joint presentation paired a presenter that described a 3rd line antiretroviral therapy (ART) program with a second country’s team that evaluated ART drug resistance patterns.

## THE HEALING POWER OF ART

The pandemic highlighted the importance of holistic health and human connection, as well as that of novel means of communication such as art as a conduit to well-being [10-12]. In addition to a classic open call for scientific and programmatic abstracts, we introduced a call for visual art pieces from Foundation patients and staff to incorporate the epistemological processes of artistic creativity and the logical processes of scientific research in the Symposium. We encouraged attendees to engage with the artists through this platform and vote on favourite submissions from each category (photography, jewellery, drawings/paintings, sculpture, textiles & crafts).

The inclusion of artwork transformed the Symposium into an opportunity to discuss the wellness benefits of art, especially considering the negative mental health impact of the pandemic on patients and frontline workers. Additionally, it enabled the engagement of HIV-positive youth, highlighted the different cultures across the network and helped foster a sense of belonging to an international community.

To showcase the relevance of art therapy in unleashing the creative imagination of children with chronic illnesses, the opening day showcased an art therapist who set the tone for the intention and power of marrying art and medicine. Additionally, short videos highlighting patients and staff artworks were presented daily. Artists from across the network talked about art in their personal and professional lives, highlighting the myriad of strengths (income generation, mindfulness, advocacy) that art can bring to life. Frontline workers often disclosed their personal use of art as an emotional outlet for coping and resilience, enabling them to develop a robust sense of self and hope as they dealt with pandemic-generated anxieties.

## PARTICIPATION

**Figure 1:**
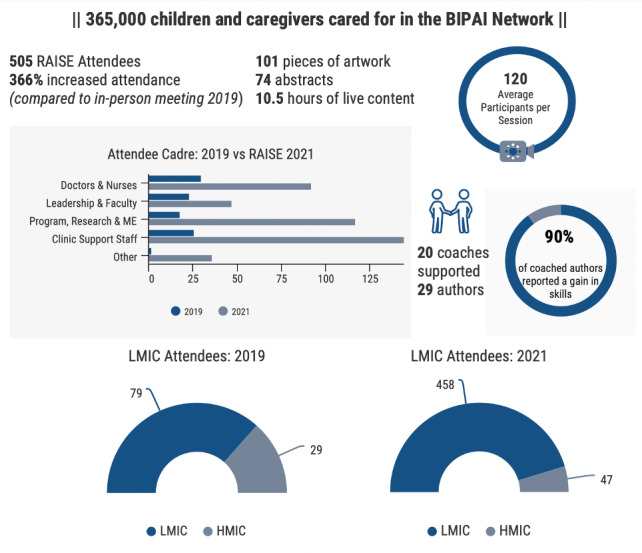
RAISE by the numbers. The RAISE symposium reached an increased number of participants compared to a prior BIPAI Network meeting, with an increased proportion of programmers, research and monitoring and evaluation (ME) colleagues. LMIC attendance increased from 73 to 90% of total participants and represented an increase of 379 LMIC attendees.

Over 500 participants from more than 20 countries registered, with an average of 49 participants per Foundation, 47 participants from TCH and BCM, and 19 external participants. (**Figure 1**) The live program averaged 120 participants per session, though this is an underestimate, as multiple participants often joined from a single account. To maximize the number of participants and broaden inclusivity further during the Symposium, we offered live interpretation in both Spanish and Romanian.

## REFLECTIONS AND LESSONS LEARNED

Combining many professional cadres and managing over 500 international registrants while trying to be inclusive and sensitive to different sites’ needs was ambitious. It required the work of many experienced professionals, commitment to sustained capacity enhancement, and meticulous coordination. To achieve this goal, the personal knowledge of the settings and beneficiaries proved fundamental. Similarly, pursuing an interdisciplinary program combining art with research sessions was bold and required a careful balance. The outcome was worthwhile, strengthening community building as witnessed by the unprecedented participation, positive feedback and increased enthusiasm and staff involvement in CoP activities following the Symposium.

We recognise that the organizational success and management strategy were partly due to the context of the pandemic, which allowed the redistribution of new tasks in the staff previously engaged in field activities. Hence, we tried to document, monitor, and evaluate in real time each step of the planning through notes, dynamic templates, and feedback surveys. This effort led to the creation of a vast archive of ready-to-use toolkits for replication.

Although we tried to anticipate most of the problems associated with the novelty of the event and the use of virtual platforms, all exacerbated by the difficulties of working remotely, internet access remained a challenge in the light of contextual events (eg, civil unrests in Eswatini and nation-wide connectivity problems in Tanzania) though not to a degree that impeded the Symposium or appreciably impacted its outcome.

The RAISE Symposium celebrated holistic health, innovation, and teamwork; its interdisciplinary approach, easily accessible virtual format, and culture of scholarship challenged the norms of traditional conferences and served as a means of inclusion to balance participation inequalities in meetings. By sharing our experience, we hope to inspire international organizations to seize the moment and re-think educational objectives and mentoring strategies for the long-term career accomplishments of LMIC staff. Thinking holistically about the lives of our colleagues around the world, rising to emerging needs and being generous with both time and resources will continue to push the needle closer to the goal of global health equity.
